# PHYTOCHROME INTERACTING FACTOR 7 is important for early responses to elevated temperature in Arabidopsis seedlings

**DOI:** 10.1111/nph.16316

**Published:** 2019-11-29

**Authors:** Anne-Sophie Fiorucci, Vinicius Costa Galvão, Yetkin Çaka Ince, Alessandra Boccaccini, Anupama Goyal, Laure Allenbach Petrolati, Martine Trevisan, Christian Fankhauser

**Affiliations:** ^1^ Faculty of Biology and Medicine Centre for Integrative Genomics University of Lausanne Génopode Building Lausanne CH‐1015 Switzerland

**Keywords:** *Arabidopsis thaliana*, auxin, PIF4, PIF7, thermomorphogenesis

## Abstract

- In response to elevated ambient temperature *Arabidopsis thaliana* seedlings display a thermomorphogenic response that includes elongation of hypocotyls and petioles. Phytochrome B and cryptochrome 1 are two photoreceptors also playing a role in thermomorphogenesis. Downstream of both environmental sensors PHYTOCHROME INTERACTING FACTOR 4 (PIF4) is essential to trigger this response at least in part through the production of the growth promoting hormone auxin.
- Using a genetic approach, we identified PHYTOCHROME INTERACTING FACTOR 7 (PIF7) as a novel player for thermomorphogenesis and compared the phenotypes of *pif7* and *pif4* mutants. We investigated the role of PIF7 during temperature‐regulated gene expression and the regulation of PIF7 transcript and protein by temperature.
- Furthermore, *pif7* and *pif4* loss‐of‐function mutants were similarly unresponsive to increased temperature. This included hypocotyl elongation and induction of genes encoding auxin biosynthetic or signalling proteins. PIF7 bound to the promoters of auxin biosynthesis and signalling genes. In response to temperature elevation *PIF7* transcripts decreased while PIF7 protein levels increased rapidly.
- Our results reveal the importance of PIF7 for thermomorphogenesis and indicate that PIF7 and PIF4 likely depend on each other possibly by forming heterodimers. Elevated temperature rapidly enhances PIF7 protein accumulation, which may contribute to the thermomorphogenic response.

## Introduction

Ambient temperature influences plants in numerous ways. Their distribution, phenology, defence capacity, growth and development are altered by modest changes in average temperature (Quint *et al.*, 2016; Gangappa *et al.*, 2017; Lau *et al.*, 2018; Casal & Balasubramanian, 2019). In response to mild temperature elevation Arabidopsis displays a number of growth and developmental responses known as thermomorphogenesis, which include accelerated flowering, hypocotyl and petiole elongation, a reduction of the stomatal index and leaf hyponasty (Quint *et al.*, 2016; Casal & Balasubramanian, 2019). Some of these responses improve the cooling capacity of Arabidopsis rosettes, which is likely important for plants to cope with increased temperature (Crawford *et al.*, 2012).

Thermomorphogenesis and photomorphogenesis are similar at different levels. This is particularly obvious when comparing shade and elevated temperature responses (Legris *et al.*, 2017). In both cases environmental sensing depends at least in part on the photoreceptors phytochrome B (phyB) and cryptochrome 1 (cry1) (Jung *et al.*, 2016; Legris *et al.*, 2016; Ma *et al.*, 2016; Pedmale *et al.*, 2016; Casal & Balasubramanian, 2019). Other signalling components including ELONGATED HYPOCOTYL 5 (HY5), CONSTITUTIVELY PHOTOMORPHOGENIC 1 (COP1) and DE‐ETIOLATED 1 (DET1), which were initially identified for their role in light responses, also play an important role in thermomorphogenesis (Delker *et al.*, 2014; Gangappa & Kumar, 2017; Park *et al.*, 2017).

PHYTOCHROME INTERACTING FACTOR 4 (PIF4) is an essential component for high temperature response under most tested conditions, while the role of PIF1, PIF3 and PIF5 is minor (Koini *et al.*, 2009; Stavang *et al.*, 2009; Nomoto *et al.*, 2012; Zhu *et al.*, 2016; Qiu *et al.*, 2019). PIF4 is inhibited by phyB and cry1 (Ma *et al.*, 2016; Qiu *et al.*, 2019), while PIF4 function depends on HEMERA that regulates both PIF4 abundance and its trans‐activating potential (Qiu *et al.*, 2019). A key role of PIF4 is to induce expression of auxin biosynthetic and signalling genes ultimately leading to hypocotyl elongation (Franklin *et al.*, 2011; Sun *et al.*, 2012; Raschke *et al.*, 2015). Hypocotyl elongation in response to shade and temperature elevation also depends on other phytohormones including gibberellic acid (GA) and brassinosteroids (BR) (Quint *et al.*, 2016; Legris *et al.*, 2017; Casal & Balasubramanian, 2019). BR acts in the hypocotyl while auxin biosynthesis mainly occurs in cotyledons before being transported to the hypocotyl to promote elongation (Stavang *et al.*, 2009; Oh *et al.*, 2012; Kohnen *et al.*, 2016; Procko *et al.*, 2016; Ibanez *et al.*, 2018; Martinez *et al.*, 2018; Bellstaedt *et al.*, 2019).

Given the overlap of signalling components regulating temperature and shade responses and the central role of PHYTOCHROME INTERACTING FACTOR 7 (PIF7) in the phyB‐mediated neighbour proximity response, we decided to test whether PIF7 is required for elevated temperature‐induced growth responses.

## Materials and Methods

### Plant material

*Arabidopsis thaliana* Columbia (Col‐0) ecotype was used. The mutants *phyB‐9* (Neff *et al.*, 1998), *cry1‐304* (Mockler *et al.*, 1999), *yuc2yuc5yuc8yuc9* (Nozue *et al.*, 2015), *pif4‐101*, *phyBpif4* (Lorrain *et al.*, 2008), *phyBpif7* (Galvao *et al.*, 2019), *phyB‐9pif4‐101pif5-3‐pif7‐1* (Goyal *et al.*, 2016), *pif4‐101pif5‐3pif7‐1* (de Wit *et al.*, 2015), *pif7‐1* and *pif7‐2* (Leivar *et al.*, 2008), were previously characterized. The transgenic PIF7‐HA line (*pif7‐2*/*pPIF7::PIF7‐3HA‐tPIF7*) was previously described (Galvao *et al.*, 2019). Furthermore, *cry1‐304phyB‐9*, *pif4‐101pif7‐2, cry1‐304pif4‐101pif5‐3* and *cry1‐304pif4‐101pif5‐3pif7‐1*, *yuc2yuc5yuc8*, *yuc2yuc5yuc9*, *yuc2yuc8yuc9* and *yuc5yuc8yuc9* were generated by crosses and confirmed by genotyping using oligonucleotides listed in the Supporting Information Table S1. The *yuc* alleles are as in Nozue *et al.* (2015).

### Phenotypic characterization and growth conditions

Seed sterilization and stratification, plant growth and light conditions were described previously (de Wit *et al.*, 2015; Kohnen *et al.*, 2016). Long‐day (LD) or short‐day (SD) photoperiods correspond to 16 h light : 8 h dark or 8 h light : 16 h dark, respectively, with *c*. 120 µmoles m^−2^ s^−1^ of photosynthetically active radiation (PAR) in LD and SD. For hypocotyl elongation measurements, seeds were sown on sterile nylon meshes on the growth media. Seedlings were grown on vertical plates in an incubator (Model AR‐22L; CLF Plant Climatics, Wertingen, Germany) for 4 d at 21°C. High temperature treatment (28°C) started on day 5 at ZT2. For picloram (Sigma‐Aldrich, Steinheim, Germany, P5575) treatment, nylon meshes were transferred on day 5 before the temperature shift to half strength MS medium with the indicated picloram concentration (0.1% dimethyl sulphoxide (DMSO) for Mock). Seedlings imaging and measurements were described previously (de Wit *et al.*, 2018). Petiole measurements were performed as described (de Wit *et al.*, 2015). Following 14 d in a LD growth room, plants were transferred to AR‐22L incubators and acclimated for 1 d to constant 21°C (LD). The next morning (ZT3), temperature in one incubator was shifted to constant 28°C. Petiole length of leaf 3 was measured after 3 d of treatment.

### RNA isolation and quantitative RT‐PCR

RNA isolation and reverse transcription quantitative polymerase chain reaction (RT‐qPCR) reactions were performed as previously described (Kohnen *et al.*, 2016). Oligonucleotides are listed in Table S1.

### ChIP‐qPCR

Briefly, 6‐d‐old PIF7‐HA (Galvao *et al.*, 2019) seedlings grown in LD at 21°C were either kept at 21°C or shifted at ZT2 to 28°C for 2 h before harvesting in liquid nitrogen. Chromatin extraction was performed as described previously (Bourbousse *et al.*, 2018) except that samples were crosslinked only with formaldehyde. Immunoprecipitation was performed as described previously (Gendrel *et al.*, 2005) using an anti‐HA antibody (Santa Cruz Biotechnology, Inc., Dallas, TX, USA; sc‐7392 X). The qPCR was done in triplicates on input and immunoprecipitated DNA. Oligonucleotides are listed in Table S1.

### Western‐blot analysis

Total protein extracts from PIF7‐HA seedlings were obtained as previously described (Galvao *et al.*, 2019). For PIF4 Western‐blot 20–25 seedlings were collected in liquid nitrogen and proteins extracted in 90 µl extraction buffer (100 mM Tris‐HCl pH 6.8, 5% SDS, 20% glycerol, 80 µM MG132, 20 mM DTT, 1× protease inhibitor cocktail (P9599; Sigma‐Aldrich), 1 mM bromophenolblue), boiled at 95°C for 5 min and centrifuged for 2 min. Protein samples were separated on 4–20% Mini‐Protean TGX gels (Bio‐Rad, Hercules, CA, USA) and blotted on nitrocellulose membrane (Bio‐Rad) using Turbo transfer system (Bio‐Rad). Membranes were blocked with 5% milk overnight at 4°C for αPIF4, and 1 h at room temperature for αHA, before probing with anti‐HA coupled with horseradish peroxidase (HRP) (Roche, Mannheim, Germany; Cat. 12013819001), polyclonal H3 (1 : 2000; Abcam, Cambridge, UK; Cat. no. 1791), polyclonal PIF4 (1 : 3000, Abiocode R2534‐4) or DET3 (1 : 20 000) antibodies. HRP‐conjugated anti‐rabbit was used as secondary antibody. Chemiluminescence signal were obtained with Immobilon Western Chemiluminescent HRP Substrate (Millipore, Merck KGaA, Darmstadt, Germany) on an ImageQuant LAS 4000 mini (GE Healthcare, Buckinghamshire, UK). Relative intensities correspond to the average of HA/H3 of six biological replicates obtained with imagej (https://imagej.nih.gov/ij/).

### Yeast two‐hybrid assay

*PIF7* and *PIF4* full length coding sequences were cloned into the pGBKT7 and pGADT7 vectors (Clontech, Mountain View, CA, USA). After co‐transformation of yeast strain TATA (Hybrigenics, Paris, France) and selection of transformants, serial cell suspensions were spotted on synthetic drop‐out medium lacking leucine and tryptophan (SD‐LW) and plates were put at 30°C for 2 d. A β‐galactosidase assay was performed directly on yeast spots as previously described (Duttweiler, 1996).

### Statistical analysis

We performed two‐way analysis of variance (ANOVA) (aov) and computed Tukey's Honest Significance Differences (HSD) test (agricolae package) with default parameters using R software (https://www.r-project.org/).

## Results

### The thermomorphogenic response depends on PIF7

**Figure 1:**
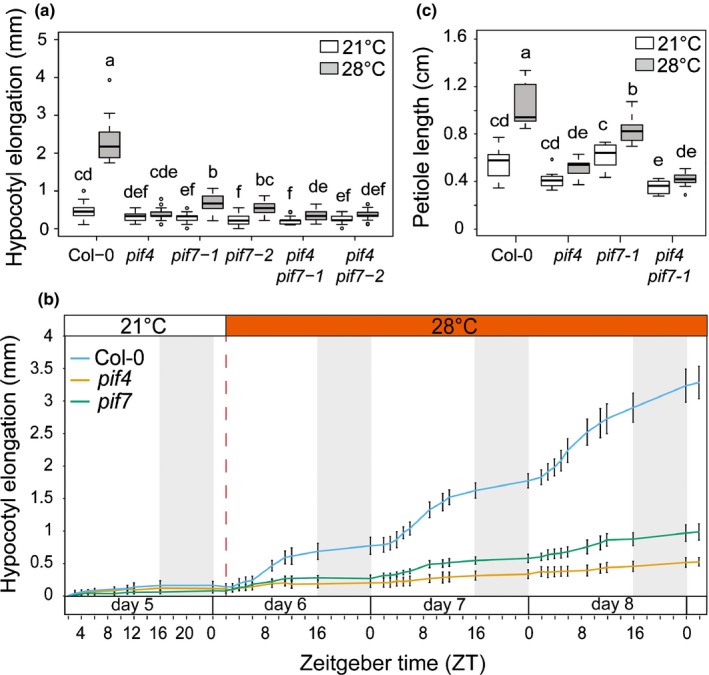
Thermomorphogenic response requires both PIF4 and PIF7 for hypocotyl and petiole elongation in Arabidopsis. (a) Hypocotyl elongation of wild‐type (Col‐0) and *pif* mutants grown in long days (LDs) at 21°C for 4 d then either kept at 21°C or transferred to 28°C (at ZT2 on day 5) for three additional days. Elongation during the last 3 d is indicated. Different letters indicate significant difference (two‐way ANOVA with Tukey's HSD test, *P* < 0.05, *n* > 25). (b) High‐resolution growth analysis of Col‐0, *pif4‐101*, and *pif7‐1* seedlings. Hypocotyl elongation from LD‐grown seedlings (21°C) was measured from time‐lapse images with indicated intervals starting from ZT0 on day 5. The red dashed line indicates start of 28°C treatment at ZT2 on day 6. The grey zone represents the dark period. Data represent means ± 2 SE; *n* > 8. (c) Petiole lengths (leaf 3) of Col‐0 and *pif* mutants grown in LD at 21°C for 15 d then either kept at 21°C or transferred to 28°C for three additional days. Different letters indicate significant difference (two‐way ANOVA with Tukey's HSD test, *P* < 0.05, *n* = 10). For (a) and (c) the horizontal bar represents the median, boxes extend from the 25^th^ to the 75^th^ percentile, while whiskers extend to 1.5 times the interquartile range of the lower and upper quartiles, respectively, outliers are indicated with circles.

We analysed the thermomorphogenic response in 4‐d‐old seedlings grown under LDs that were either kept at 21°C or transferred to 28°C for three additional days. We used this shift protocol to allow us to investigate the early response to increasing temperature. Consistent with previous reports (Koini *et al.*, 2009; Stavang *et al.*, 2009), wild‐type Col‐0 (WT) hypocotyl elongated robustly at 28°C while *pif4* was largely unresponsive (Fig. 1a). The phenotype of both tested *pif7* alleles was slightly less severe than *pif4* while *pif4pif7* was similar to *pif4* (Fig. 1a). We also analysed the thermomorphogenic hypocotyl elongation response in SDs and found that *pif7* like *pif4* was largely unresponsive to temperature elevation (Fig. S1). We conclude that PIF7 is required for elevated ambient temperature‐induced hypocotyl elongation irrespective of day length and conducted all subsequent experiments in LDs because in nature higher temperatures are more common when days get long.

To determine whether the phenotype observed after 3 d reflects a similar defect in the growth pattern we followed growth kinetics of the WT, *pif4* and *pif7*. Elevated temperature enhanced growth during the day while growth at night was limited in both conditions (Fig. 1b) (Park *et al.*, 2017). Enhanced elongation triggered at 28°C during the first day of treatment depended on *PIF4* and *PIF7* (Fig. 1b). Consistent with the phenotype observed after 3 d, *pif7* grew slightly more than *pif4* during the next 2 d (Fig. 1b). We conclude that in response to temperature elevation growth during the day depends on PIF4 and PIF7. In constant light and LD, PIF4 controls day growth downstream of phyB and cry1 (Ma *et al.*, 2016; Qiu *et al.*, 2019). The importance of PIF7 in warm LD (Fig. 1a,b) prompted us to measure hypocotyl growth of *phyBpif* and *cry1pif* mutant combinations. Both *phyB* and *cry1* mutants showed robust temperature‐induced elongation while the *phyBcry1* double mutant was unresponsive suggesting that both photoreceptors are crucial for temperature‐controlled hypocotyl elongation (Fig. S2a). However, we note that *cry1phyB* double mutant had very long hypocotyls at 21°C possibly limiting further elongation at 28°C. As observed previously (Qiu *et al.*, 2019), *pif4* partially suppressed *phyB* (Fig. S2a,b). However, *phyBpif7* had shorter hypocotyls than *phyBpif4*, both at 21°C and 28°C, highlighting the importance of PIF7 for phyB repressed hypocotyl elongation (Fig. S2a,b). The *phyB* phenotype was almost totally suppressed in *phyBpif4pif5pif7* (Fig. S2b). Consistent with the dominant function of PIF4 downstream of cry1 (Ma *et al.*, 2016), *pif4pif5* was epistatic over *cry1* with no further suppression observed in *cry1pif4pif5pif7* (Fig. S2c). We conclude that PIF4 and PIF7 both promote hypocotyl elongation in response to increased temperature in LD, while their regulation by photoreceptors differs at least partially.

Later in development high temperature leads to petiole elongation (Koini *et al.*, 2009), which we analysed in young rosettes that were either maintained at 21°C or transferred for 3 d to 28°C. Elevated temperature‐induced petiole elongation was most affected in *pif4*, but also impaired in *pif7* and the response of *pif4pif7* was very similar to *pif4* (Fig. 1c). Taken together our results indicate that PIF7 is almost as important as PIF4 for thermomorphogenic growth responses.

### PIF7 controls temperature‐induced expression of ‘auxin’ genes

**Figure 2:**
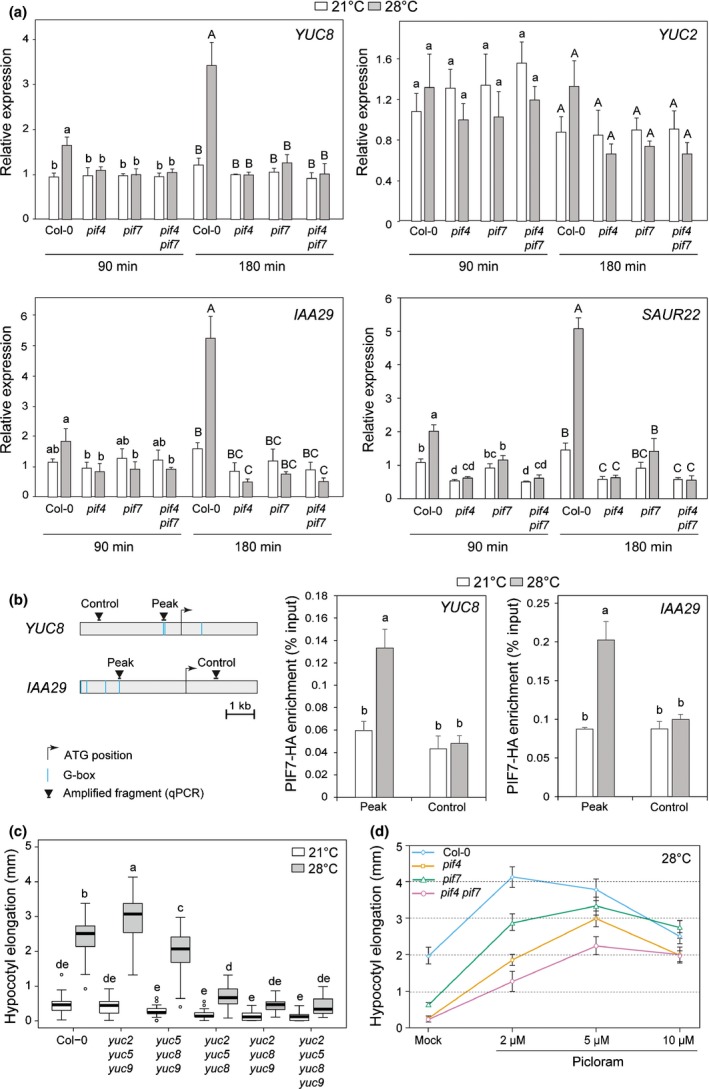
PIF4 and PIF7 regulate the auxin pathway during thermomorphogenesis in Arabidopsis. (a) Relative expression of auxin biosynthesis (*YUC2* and *YUC8*) and auxin response (*IAA29* and *SAUR22*) genes in 5‐d‐old Col‐0 and *pif* mutants either kept at 21°C or transferred to 28°C at ZT2; samples at 21 and 28°C were harvested at the same ZT. Gene expression values were calculated as fold induction relative to a Col‐0 sample at 21°C, *t* = 90 min. *n* = 3 (biological) with three technical replicas for each RNA sample. Data are means, ± 2 SE. Different letters indicate significant differences within timepoints (*P* < 0.05). (b) PIF7‐HA binding to the promoter of *YUC8* and *IAA29* evaluated by ChIP‐qPCR in 6‐d‐old seedlings either kept at 21°C or transferred for 2 h to 28°C at ZT2. Input and immunoprecipitated DNA were quantified by qPCR using primers shown on the schematic representation of the genes with ‘Peak’ indicating where PIF4 binding was identified before (left). PIF7‐HA enrichment is presented as IP/Input and error bars show standard deviation from three technical replicas. Different letters indicate significant differences (*P* < 0.05). Data from one representative experiment are shown. (c) Hypocotyl length of wild‐type (Col‐0) and *yuc* mutants grown in long day (LD) at 21°C for 4 d then kept at 21°C or transferred to 28°C for three additional days. Growth during the last 3 d is indicated. The horizontal bar represents the median, boxes extend from the 25^th^ to the 75^th^ percentile, while whiskers extend to 1.5 times the interquartile range of the lower and upper quartiles, respectively, outliers are indicated with circles. Different letters indicate significant difference (two‐way ANOVA with Tukey's HSD test, *P* < 0.05 *n* > 25). (d) Hypocotyl elongation at 28°C of Col‐0 and *pif* mutants in response to indicated concentrations of exogenously applied synthetic auxin, picloram. Seedlings were grown and measured as indicated in (a) picloram was applied at the time of transfer to 28°C. Data represent means ± 2 SE; *n* > 25.

PIF4 is essential to induce expression of *YUC* genes leading to higher auxin levels and growth (Sun *et al.*, 2012). Transfer to 28°C led to significantly increased expression of *YUC8* and *YUC9* (after 90 and 180 min) while *YUC2* induction was modest and not significant (Figs 2a, S3). PIF4 and PIF7 were required for temperature‐induced expression of *YUC8* and the auxin signalling genes *IAA29* and *SAUR22* (Fig. 2a), demonstrating the requirement of both phytochrome‐interacting factors (PIFs) for enhanced ‘auxin gene’ expression and growth (Figs 1, 2). To test whether PIF7 may directly control the expression of *YUC8* and *IAA29* we performed ChIP experiments using a full genomic PIF7‐HA line (Galvao *et al.*, 2019). This experiment showed that after 2 h at 28°C PIF7 was bound to the promoter of *YUC*8 and *IAA29* at a position where PIF4 binding was reported previously (Fig. 2b) (Hornitschek *et al.*, 2012; Sun *et al.*, 2012). To assess the functional importance of temperature‐induced *YUC* expression we analysed hypocotyl elongation in a *yuc2yuc5yuc8yuc9* quadruple mutant and all possible triple mutants. This experiment confirmed the importance of YUC8 and revealed a role for YUC2 in thermomorphogenesis (Fig. 2c) (Sun *et al.*, 2012). In response to a lower red to far‐red (R : FR) ratio indicative of neighbouring plants PIF7 plays a particularly important role to enhance auxin production while PIF4 also regulates the response to auxin (Nozue *et al.*, 2011; Hornitschek *et al.*, 2012; Li *et al.*, 2012; Pucciariello *et al.*, 2018). We therefore compared the sensitivity of *pif4*, *pif7* and *pif4pif7* mutants to the synthetic auxin picloram in seedlings grown at 28°C (Fig. 2d). This experiment showed that although *pif7* had a very small response to 28°C (mock) it responded like the WT to 2 µM picloram, while *pif4* had a reduced response. At higher picloram concentrations *pif7* also responded less than the WT. We note that the lower picloram response of *pif4* compared to *pif7* correlates with the growth phenotypes of the mutants after prolonged elevated temperature treatments (Figs 1, 2d).

To investigate whether PIF4 and PIF7 regulate the same process required for temperature‐induced hypocotyl elongation rather than different independently required steps, we analysed expression of hormone biosynthetic genes that were previously implicated in thermomorphogenesis (Stavang *et al.*, 2009). In our conditions expression of the BR biosynthetic gene *BRASSINOSTEROID‐6‐OXIDASE 2* (*BR6ox2*) was induced by higher temperature in WT plants but not in *pif7* and *pif4* (Figs S3, S4). Temperature‐induced expression of *CONSTITUTIVE PHOTOMORPHOGENIC DWARF* (*CPD*) depended more on PIF4 than PIF7 while higher expression of the gibberellic acid biosynthesis gene *GIBBERELLIN‐3‐OXIDASE 1* (*GA3OX1*) was largely independent of PIF4 or PIF7 (Figs S3, S4). Similarly, strong induction of a small heat‐shock gene (*HSP17.6B*) was unaffected in the tested *pif* mutants (Figs S3, S4). We therefore conclude that *pif4* and *pif7* show a similar temperature‐regulated gene expression pattern with a particularly obvious effect on auxin biosynthesis and response genes (Figs 2, S3, S4).

### PIF7 does not regulate PIF4 accumulation but both PIFs can interact with each other

**Figure 3:**
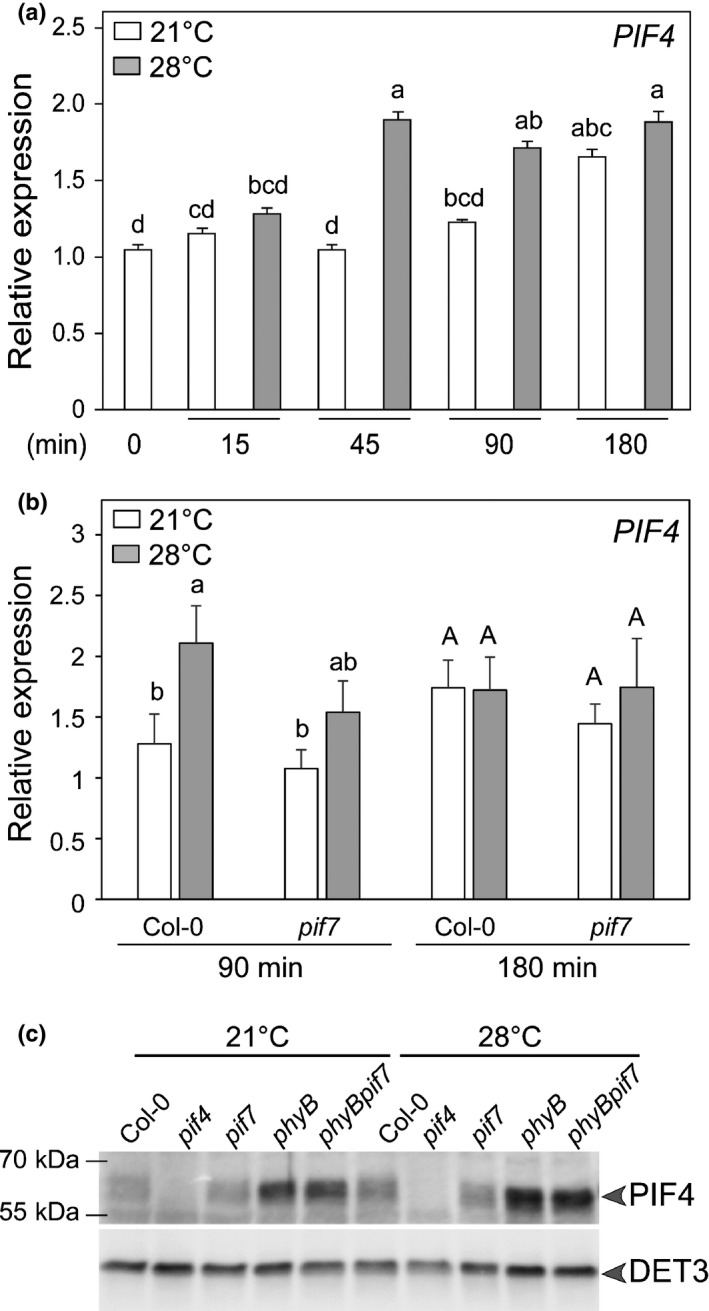
Regulation of PIF4 mRNA and protein levels in thermomorphogenesis in Arabidopsis. (a) Relative expression of *PIF4* in 5‐d‐old Col‐0 and (b) in Col‐0 and *pif7‐1* mutants. Seedlings were grown as in Fig. 2(a). Gene expression values were calculated as fold induction relative to a Col‐0 sample at 21°C, *t* = 0 (a) and *t* = 90 min (b). *n* = 3 (biological) with three technical replicas for each RNA sample. Data are means, ± 2 SE. Different letters indicate significant differences (*P* < 0.05). (c) PIF4 protein levels in the indicated genotypes detected with anti‐PIF4 antibody from total protein extracts after 3 h of 21°C and 28°C treatment in 5‐d‐old long day‐grown seedlings treated at ZT2. DET3 was used as a loading control.

The central importance of PIF4 for thermomorphogenesis prompted us to determine whether PIF7 is required for PIF4 accumulation. We compared *PIF4* mRNA levels in the WT and *pif7* at 21°C and 28°C and did not detect a major effect of PIF7 on *PIF4* expression (Fig. 3a,b). PIF4 protein levels at 21°C and the slight increase observed after 3 h at 28°C were similar in the WT and *pif7* (Fig. 3c). Consistent with phyB promoting PIF4 degradation (de Lucas *et al.*, 2008), we detected high PIF4 levels in *phyB* and *phyBpif7* at 21°C and PIF4 levels increased at 28°C independently of PIF7 (Fig. 3c). Alternatively, PIF4 and PIF7 might be both required for thermomorphogenesis because they work as a heterodimer to regulate gene expression (Fig. 2). We used the yeast two‐hybrid assay to determine whether both proteins can interact and found that PIF4 and PIF7 form homodimers and heterodimers in yeast (Fig. S5). We conclude that the strong thermomorphogenic phenotype of *pif7* cannot be explained by lower PIF4 protein levels but may be due to PIF4/PIF7 heterodimer‐mediated gene expression (Figs 2, S5).

### PIF7 protein levels increase rapidly upon transfer to 28°C

**Figure 4:**
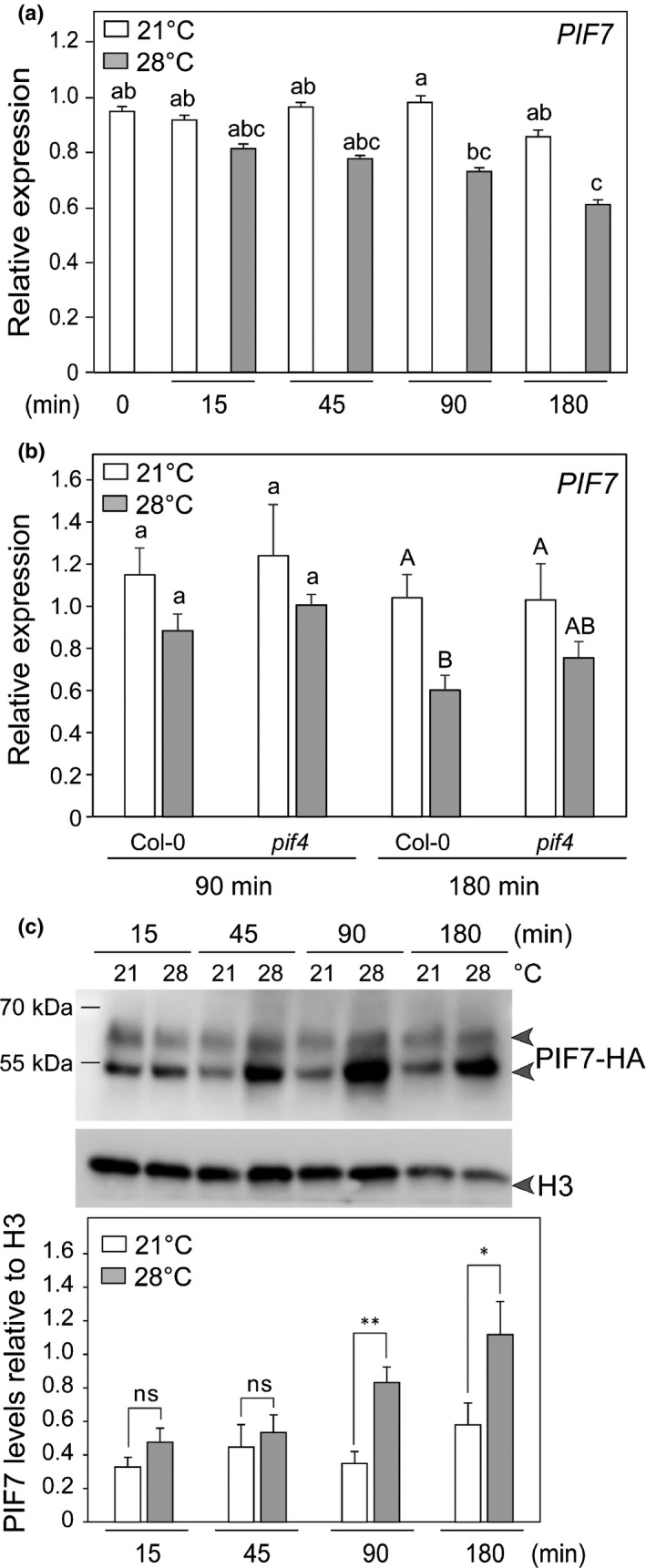
Regulation of PIF7 mRNA and protein levels in thermomorphogenesis in Arabidopsis. (a) Relative expression of *PIF7* in 5‐d‐old Col‐0 and (b) in Col‐0 and *pif4* mutant. Seedlings were grown as in Fig. 2(a). Gene expression values were calculated as fold induction relative to a Col‐0 sample at 21°C, *t* = 0 (a) and *t* = 90 min (b). *n* = 3 (biological) with three technical replicas for each RNA sample. Data are means, ± 2 SE. Different letters indicate significant differences (*P* < 0.05). (c) PIF7‐HA protein levels detected with an anti‐HA antibody from total protein extract after indicated time points at 21°C and 28°C in 5‐d‐old long day‐grown seedlings treated at ZT2. The HA signal was quantified and normalized to H3 signal (*n* = 6). Data are means, ±SE. Asterisks indicate significant (*P* values) between 28°C and 21°C samples at a given timepoint (Student's *t*‐test: * < 0.05; ** < 0.01), ns, nonsignificant.

The requirement of PIF7 for rapid temperature‐induced changes in gene expression and hypocotyl elongation (Figs 1, 2) suggested that PIF7 function and/or accumulation might be temperature‐regulated. Upon transfer to 28°C *PIF7* transcript levels declined in the WT while in *pif4* we observed a similar but not significant reduction (Fig. 4a,b). To analyse PIF7 protein we used a PIF7‐HA line (Galvao *et al.*, 2019) and found that in contrast to *PIF7* RNA, PIF7 protein levels increased significantly 90 min after the transfer to 28°C (Fig. 4c). As observed previously PIF7 was present as two major isoforms (Li *et al.*, 2012) (Fig. 4c). Transfer to 28°C specifically led to increased abundance of the faster migrating isoform (Figs 4c, S6). We propose that temperature‐induced PIF7 levels may contribute to enhanced PIF7 activity required for rapid thermomorphogenic responses.

## Discussion

PIF4 was believed to have a uniquely important function in thermomorphogenesis (Koini *et al.*, 2009; Sun *et al.*, 2012; Ma *et al.*, 2016; Casal & Balasubramanian, 2019; Qiu *et al.*, 2019). Our work shows that in seedlings the role of PIF7 is almost as important as PIF4 (Figs 1, 2, S1, S2). However, upon prolonged growth at 28°C we observed a slightly greater growth response in *pif7* than *pif4* (Fig. 1b,c). In addition, we found that at 28°C *pif4* responds less to picloram than *pif7* (Fig. 2c). A greater role of PIF4 than PIF7 in controlling auxin responsiveness may explain the small phenotypic difference between both *pif* mutants. Our data on thermomorphogenesis reveals interesting similarities and differences with the shade avoidance response. A reduction of the R : FR ratio indicative of neighbour proximity leads to auxin synthesis that primarily depends on PIF7 (Li *et al.*, 2012). Our data suggests that during thermomorphogenesis PIF4 and PIF7 are similarly important to promote auxin biosynthesis (Fig. 2a). PIF4 and PIF5 rather than PIF7 have been implicated in the control of auxin sensitivity (Nozue *et al.*, 2011; Hornitschek *et al.*, 2012; Li *et al.*, 2012; Hersch *et al.*, 2014; Pucciariello *et al.*, 2018). We find that during thermomorphogenesis PIF4 also plays a more important function than PIF7 to promote auxin responsiveness (Fig. 2d). Finally, our data on temperature‐induced growth (Figs 1, 2) is consistent with a model emerging from the study of the low R : FR response with an early phase depending on auxin production (Tao *et al.*, 2008; Li *et al.*, 2012) and a prolonged response requiring PIF4‐controlled auxin sensitivity (Pucciariello *et al.*, 2018).

Several possibilities can explain the requirement of PIF4 and PIF7 for thermomorphogenesis. Each of them might control different essential steps for elevated temperature‐induced growth. Given the similar gene expression profile of *pif4* and *pif7*, this is an unlikely explanation (Figs 2, S4). However, more research is required to test this hypothesis on a larger scale and with better spatial resolution (e.g. hypocotyls vs cotyledons). Given that both single mutants and the *pif4pif7* double mutant have similar phenotypes (Figs 1, 2, S1), the function of these PIFs might depend on each other. We showed that PIF7 is not required for normal accumulation of *PIF4* transcript or PIF4 protein and *PIF7* mRNA expression is largely unaffected in *pif4* (Figs 3, 4). As bHLH transcription factors bind DNA as dimers, an attractive hypothesis is that a PIF4/PIF7 heterodimer regulates expression of target genes such as *YUC8* or *IAA29* (Fig. 2) (Hornitschek *et al.*, 2012; Li *et al.*, 2012; Sun *et al.*, 2012). Consistent with this hypothesis, PIF4 and PIF7 interact with each other when co‐expressed in mesophyll protoplasts (Kidokoro *et al.*, 2009) and in the yeast two‐hybrid assay (Fig. S5). Collectively, these findings support the PIF4/PIF7 heterodimer hypothesis during thermomorphogenesis. However, in the *phyB* mutant background *pif* mutants act additively with almost full *phyB* suppression in *phyBpif4pif5pif7* indicating that the different PIFs can act independently (Fig. S2b). Additive effects of PIF4 and PIF7 have also been observed during de‐etiolation and shade avoidance (Leivar *et al.*, 2008; de Wit *et al.*, 2016). Similarly, PIF4 and PIF7 act independently of each other to suppress cold tolerance during long days (Lee & Thomashow, 2012). We conclude that additional research is required to understand to what extent PIF4 and PIF7 activity depend on each other and how this dependency may be regulated by development or the environment.

Temperature elevation regulates *PIF7* transcript and protein levels in opposite ways with a reduction of transcript but more PIF7 protein (Fig. 4). Reducing the R : FR ratio also leads to lower *PIF7* transcripts, while PIF7 phosphorylation changes, which regulates PIF7 accumulation in the nucleus (Li *et al.*, 2012; Huang *et al.*, 2018). Photoperiod also regulates PIF7 protein and *PIF7* mRNA with higher levels of *PIF7* transcript and PIF7 protein in LD (Lee & Thomashow, 2012). In response to increasing ambient temperature PIF7 protein levels, particularly its faster isoform increased rapidly (Figs 4c, S6). We propose that the temperature‐enhanced PIF7 protein levels may contribute to the thermomorphogenic response. It will be interesting to decipher the mechanisms underlying this change in PIF7 accumulation and if/how this regulation contributes to enhanced PIF7 function at elevated temperature.

## Author contributions

Conceptualization, ASF, VCG, YCI and CF; investigation, ASF, VCG, YCI and AB; resources, AG, MT and LAP; funding acquisition, CF; writing, CF; supervision, CF. ASF, VCG and YCI contributed equally to this work.

## Supporting information

Please note: Wiley Blackwell are not responsible for the content or functionality of any Supporting Information supplied by the authors. Any queries (other than missing material) should be directed to the *New Phytologist* Central Office.
**Fig. S1** Thermomorphogenic response requires both PIF4 and PIF7 for hypocotyl elongation in short day (SD).
**Fig. S2** PIF4 and PIF7 regulate thermomorphogenic hypocotyl elongation downstream of phyB and cry1.
**Fig. S3** Relative expression of genes that were previously implicated in thermomorphogenesis.
**Fig. S4** Relative expression of temperature‐induced genes in Col‐0 and pif mutants.
**Fig. S5** PIF7 and PIF4 form homodimers and heterodimers in yeast.
**Fig. S6** Regulation of the levels of both PIF7 isoforms in thermomorphogenesis.
**Table S1** List of oligonucleotides used in this study.
