# Supplementary material for: PHYTOCHROME INTERACTING FACTOR 7 is important for early responses to elevated temperature in Arabidopsis seedlings
Source: New Phytol. 2019 Nov 29;226(1):50–8. doi: 10.1111/nph.16316 (PMC7064998; doi:10.1111/nph.16316)
Supplement: Supplementary file 1 — Please note: Wiley Blackwell are not responsible for the content or functionality of any Supporting Information supplied by the authors. Any queries (other than missing material) should be directed to the New Phytologist Central Office. Fig. S1 Thermomorphogenic response requires both PIF4 and PIF7 for hypocotyl elongation in short day (SD). Fig. S2 PIF4 and PIF7 regulate thermomorphogenic hypocotyl elongation downstream of phyB and cry1. Fig. S3 Relative expression of genes that were previously implicated in thermomorphogenesis. Fig. S4 Relative expression of temperature‐induced genes in Col‐0 and pif mutants. Fig. S5 PIF7 and PIF4 form homodimers and heterodimers in yeast. Fig. S6 Regulation of the levels of both PIF7 isoforms in thermomorphogenesis. Table S1 List of oligonucleotides used in this study. [file NPH-226-50-s001.pdf]

New Phytologist

***New Phytologist* Supporting Information**

Article title: **PHYTOCHROME INTERACTING FACTOR 7 is important for early responses to elevated temperature in Arabidopsis seedlings.**

Authors: Anne Sophie Fiorucci, Vinicius Costa Galvão, Yetkin Çaka Ince, Alessandra Boccaccini, Anupama Goyal, Laure Allenbach Petrolati, Martine Trevisan and Christian Fankhauser

Article acceptance date: 5 November 2019

The following Supporting Information is available for this article:

Fig. S1 Thermomorphogenic response requires both PIF4 and PIF7 for hypocotyl elongation in short-day (SD).

Fig. S2 PIF4 and PIF7 regulate thermomorphogenic hypocotyl elongation downstream of phyB and cry1.

Fig. S3 Relative expression of genes that were previously implicated in thermomorphogenesis.

Fig. S4 Relative expression of temperature-induced genes in Col-0 and *pif* mutants.

Fig. S5 PIF7 and PIF4 form homo- and hetero-dimers in yeast.

Fig. S6 Regulation of the levels of both PIF7 isoforms in thermomorphogenesis.

Table S1 List of oligonucleotides used in this study.

Fig. S1. Thermomorphogenic response requires both PIF4 and PIF7 for hypocotyl elongation in short-day (SD). Hypocotyl elongation of wild-type (Col-0) and *pif* mutants grown in SD (8h light, 16 hour dark) at 21°C for 4 days then either kept at 21°C or transferred to 28°C (at ZT2 on day 5) for five additional days. Elongation during the last 5 days is indicated. Different letters indicate significant difference (two-way ANOVA with Tukey's HSD test, $P < 0.05$, n > 25).

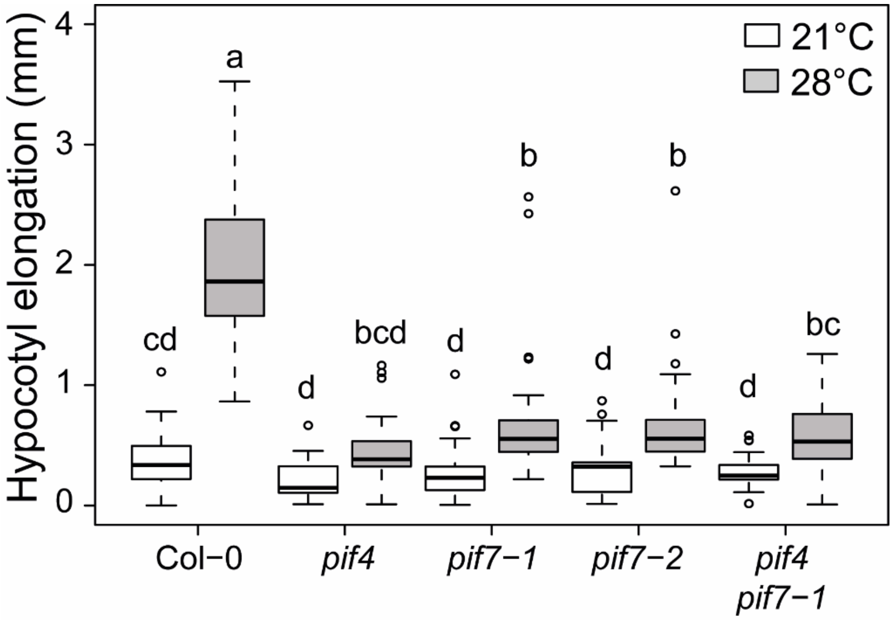

Fig. S2 PIF4 and PIF7 regulate thermomorphogenic hypocotyl elongation downstream of phyB and cry1. (a-c) Hypocotyl elongation of indicated genotypes grown in LD at 21°C for 4 days then either kept at 21°C or transferred to 28°C for three additional days. Elongation during the last 3 days is indicated. Different letters indicate significant difference (two-way ANOVA with Tukey's HSD test, $P < 0.05$, n > 25).

(a)

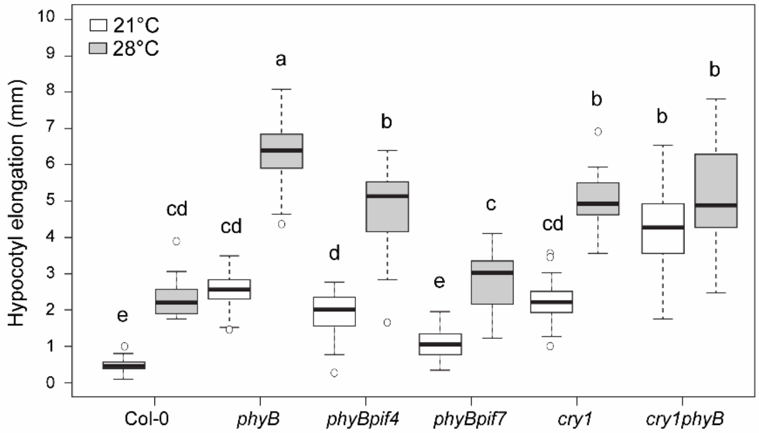

(b)

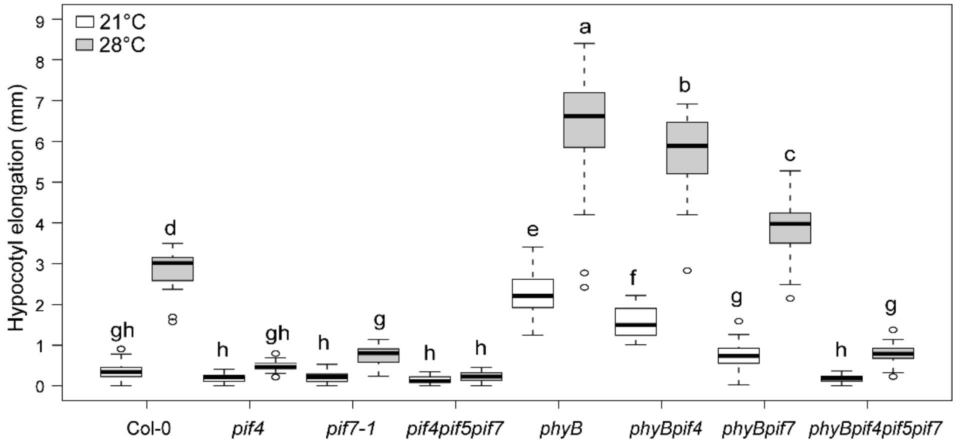

(c)

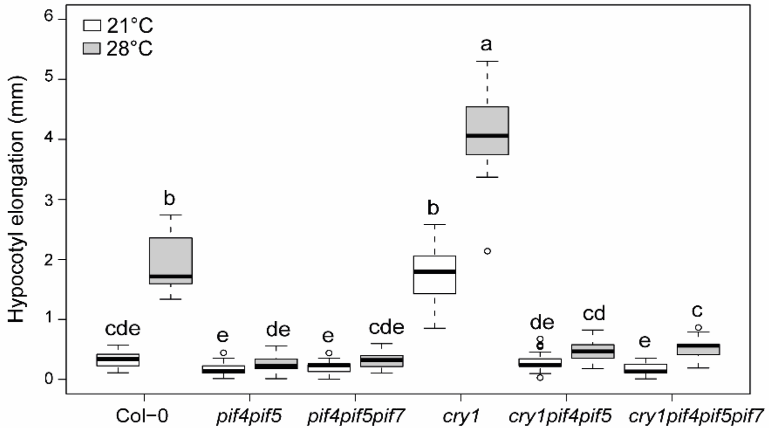

Fig. S3 Relative expression of genes that were previously implicated in thermomorphogenesis. Seedlings were grown as indicated in Fig. **2a**. Gene expression values were calculated as fold induction relative to a Col-0 sample at 21°C, t = 0. n = 3 (biological) with 3 technical replicas for each RNA sample. Data are mean, error bar indicates 2XSE. Different letters indicate significant difference (two-way ANOVA with Tukey's HSD test, *P* < 0.05).

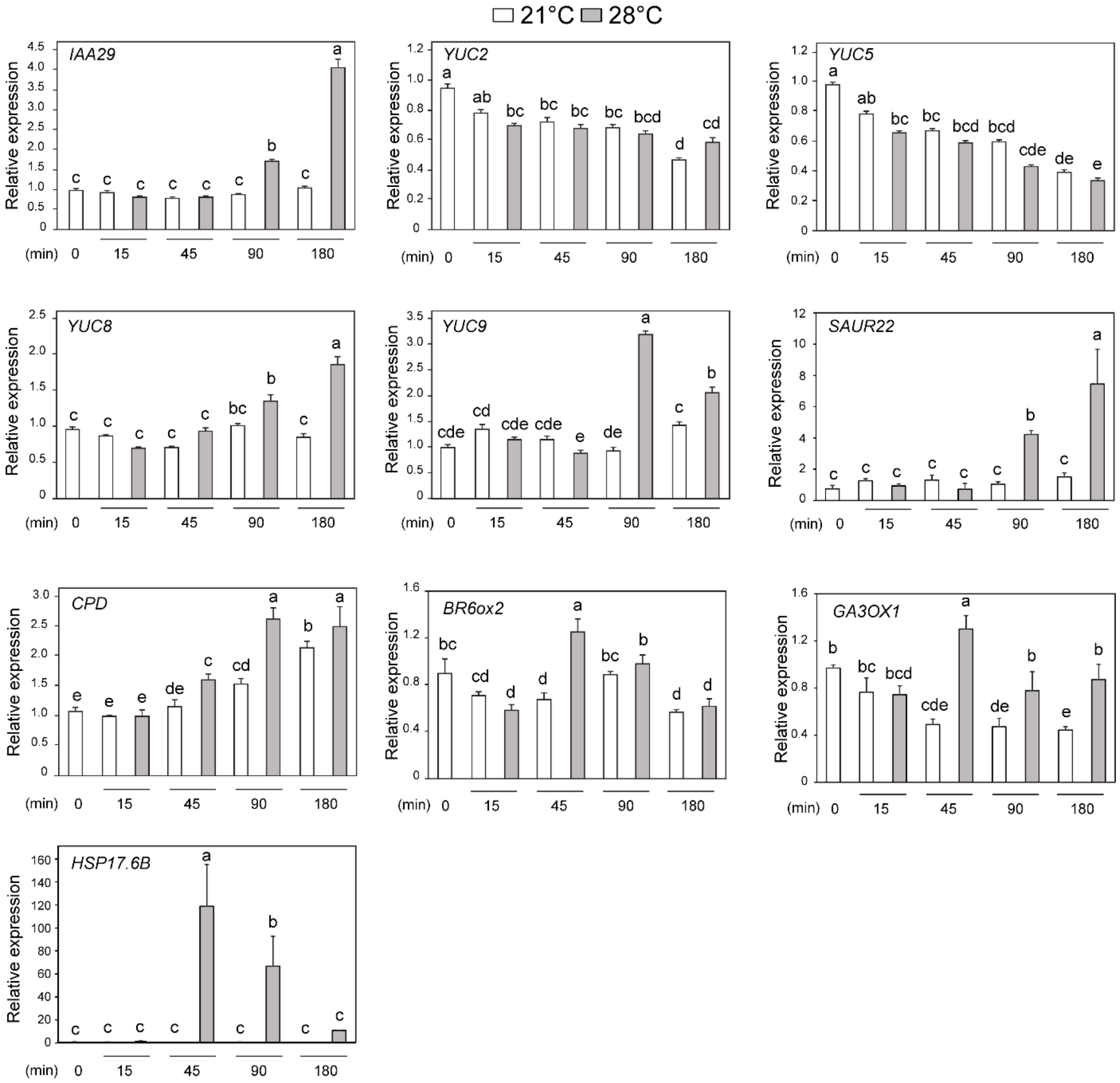

Fig. S4 Relative expression of temperature-induced genes in Col-0 and *pif* mutants. Seedlings were grown as indicated in Fig. **2a**. Gene expression values were calculated as fold induction relative to a Col-0 sample at 21°C, t = 90 min. n = 3 (biological) with 3 technical replicas for each RNA sample. Data are mean, error bar indicates 2XSE. Different letters indicate significant differences within timepoints (p<0.05).

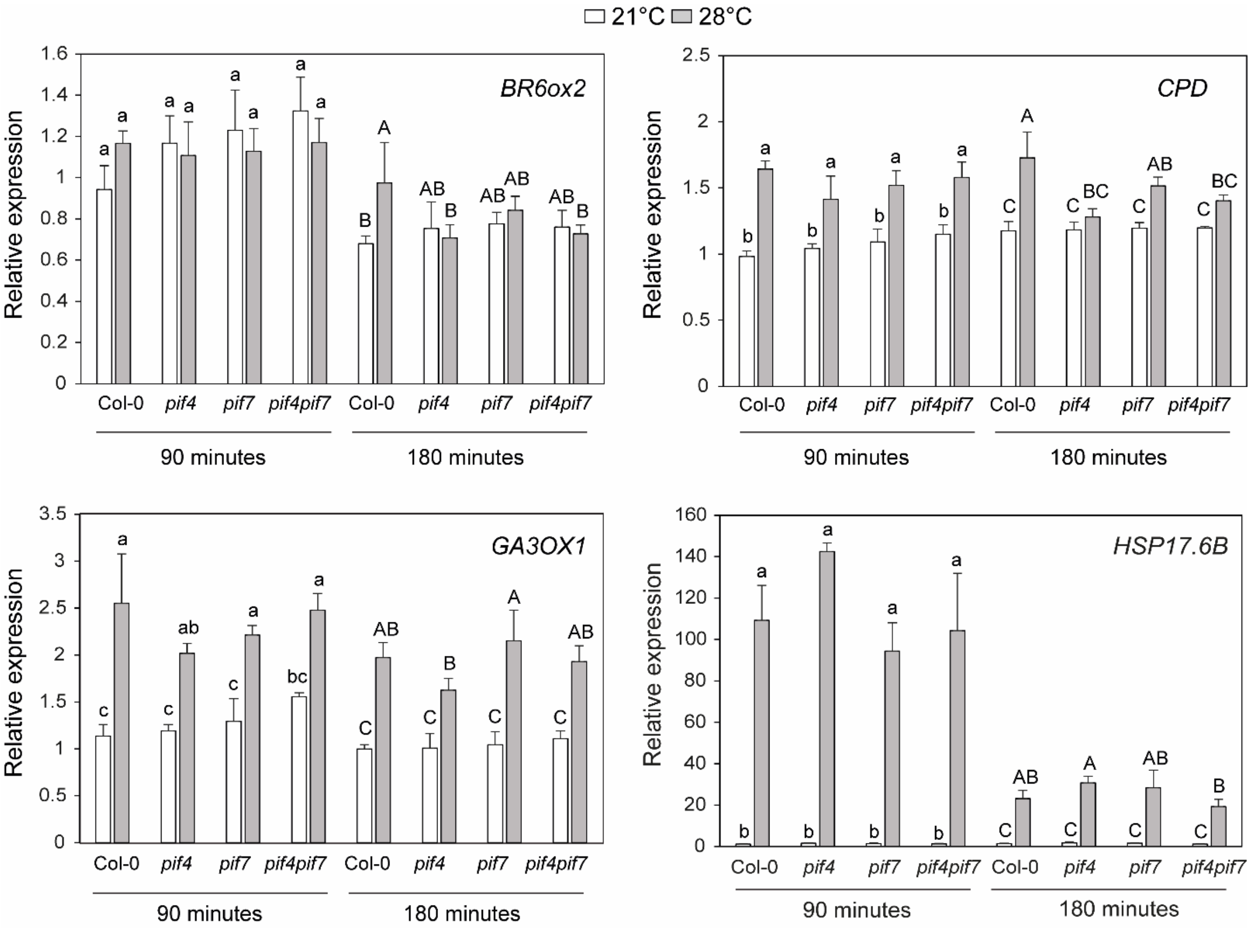

Fig. S5 PIF7 and PIF4 form homo- and hetero-dimers in yeast. Yeast two-hybrid ß-galactosidase assay testing the interactions between full-length PIF7 and PIF4 fused to either GAL4 binding domain (BD-) or GAL4 activation domain (AD-). Yeast co-transformed with the indicated vectors were spotted on SD-LW medium (10x serial dilutions from OD0.1 to OD0.001) and grown for two days at 30°C before an X-gal-containing agarose overlay. Plates were kept at 37°C in darkness and pictures were taken after 5h (all combinations with BD-PIF4) and 22h (all the others). Empty pGBKT7 and pGADT7 vectors are used as negative controls.

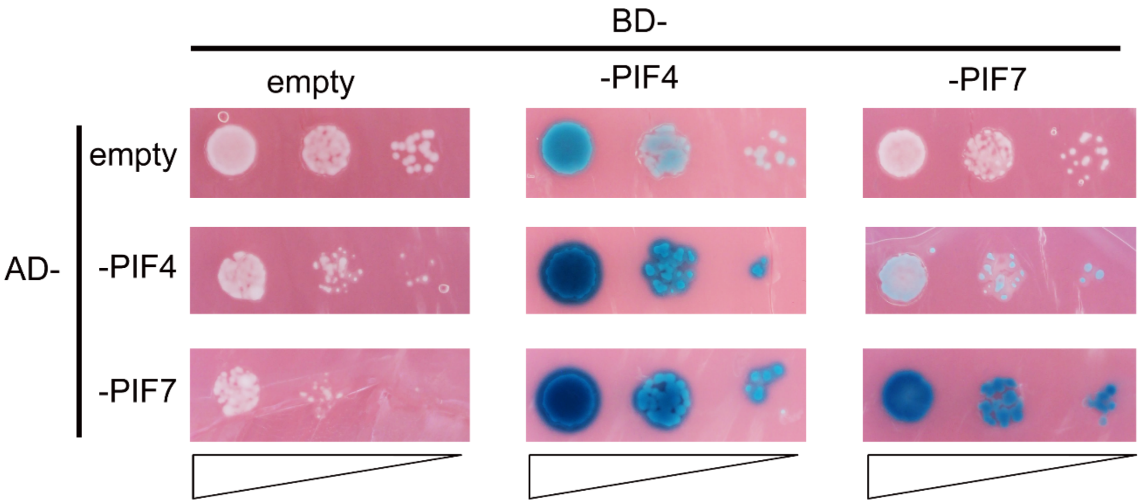

Fig. S6 Regulation of the levels of both PIF7 isoforms in thermomorphogenesis. (a) Slow-migrating and (b) fast-migrating isoforms of PIF7-HA protein detected with anti-HA antibody from total protein extracts after the indicated time points at 21°C and 28°C in 5-days-old LD-grown PIF7-HA seedlings treated at ZT2. The HA signal was quantified and normalized to H3 signal (n = 6). Data are mean, error bar indicates SE. Asterisks indicate significant difference (*p* values) between 28°C and 21°C samples at a given timepoint (Student's t-test, * < 0.05, ** < 0.01), n.s. non significant.

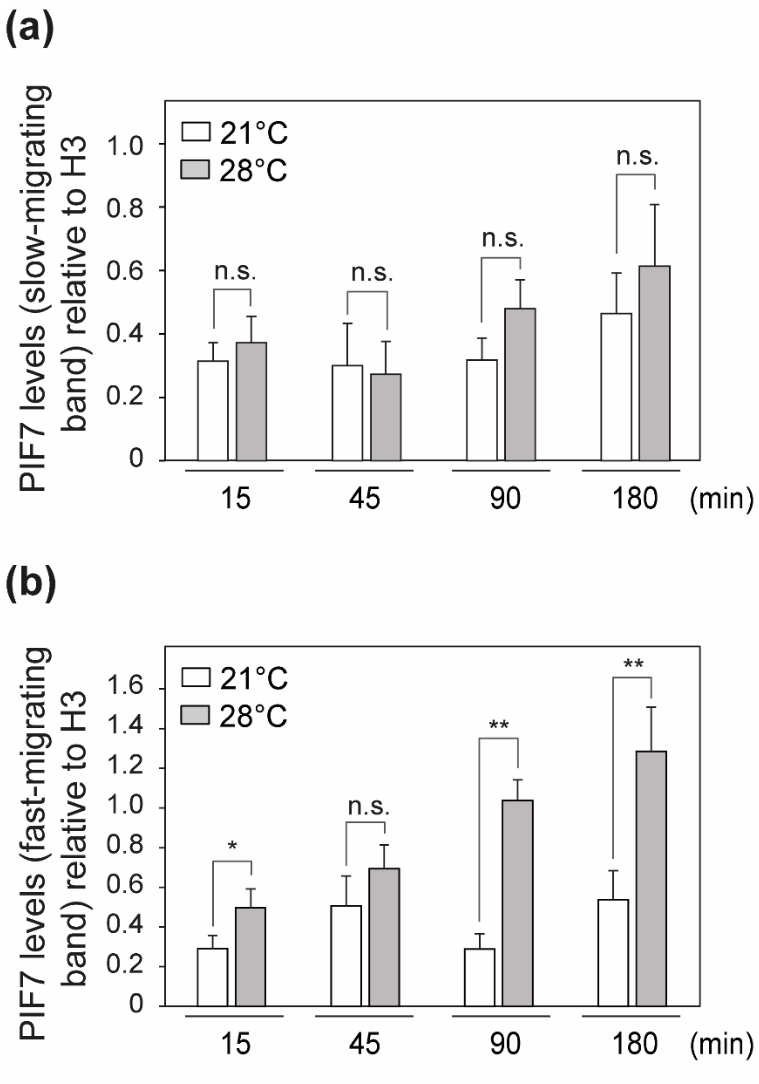

Table S1 List of oligonucleotides used in this study.

Oligos used for genotyping			
**Allele**	**Collection**	**Oligonucleotide**	**Sequence**
phyB-9	Point mutation	PB9	GTGTCTGCGTTCTCAAAACG
		B9dCAPS	GTGGAAGAAGCTCGACCAGGCTTTG
cry1-304	Deletion	CF586	GGTAGGGTTTCTAGGTGGTGGCTC
		CF587	GGTGGAAGAAGAGGAGACTCAGGG
yuc2-1	SALK_030199	oJM1845	TTCTTGCATTTTCTCGCTCTACG
		MT440	AACCCGTGGCGAGTATAATG
yuc5-3	GT6160	oJM1203	CGGACTCTAATCAAAGTCCC
		oJM1204	GGAGATTTCAAAACTAGATTTG
yuc8-1	CS110939	oJM1206	CATCCTCTCCACGTGGCTTCC
		oJM1207	GAACTGACGCTTCGTCGGGTAC
yuc9-1	SAIL_871G01	oJM1199	GCTCGGTAAGCAAAACAAAACTG
		oJM1200	GAAGGAAATGCCCAATGAGAC
pif4-101	SAIL_114_G06	SL-43	CAGACGGTTGATCATCTG
		oVCG-61	TAGCATCTGAATTTCATAACCAATCTCGATACAC
pif5-3 (pil6-1)	SALK_087012	SL-46	TCGCTCACTCGCTTACTTAC
		oVCG-56	ATTTTGCCGATTTCGGAAC
pif7-1	CS68809	SL-195	GTGGCAAGTTGGCTCTTAGG
		SL-169	TGATAGTGACCTTAGGCGACTTTTGAACGC
pif7-2	SAIL_622_G02	oASF-27	GGAGAGCCATAGAGTTGG
		oVCG-61	TAGCATCTGAATTTCATAACCAATCTCGATACAC

Oligos used for RT-qPCR			
**Target**	**Primer Efficiency**	**Oligonucleotide**	**Sequence**
UBC	1.94	UBC-F	CAGTCTGTGTGTAGAGCTATCATAGCAT
		UBC-R	AGAAGATTCCCTGAGTCGCAGTT
YSL8	2.00	YSL8-F	TCATTCGTTTCGGCCATGA
		YSL8-R	CTCAGCAACAGACGCAAGCA
PIF4	2.02	oVCG-246	TACCTCGATTTCCGGTTATGGATC
		oVCG-247	GTTGTTGACTTTGCTGTCCCGC
	1.78	SL63	TTCTCCTCCCACTTCTTCTC
		SL64	AGGTTCAGGACTGGACTTAG
PIF7	2.01	oVCG-588	GAGCAGCTCGCTAGGTACATG
		oVCG-589	GTTGTTGTTGCACGGTCTG
YUC2	1.94	MT-437	AACTCCGGGATGGAAGTTTG
		MT-438	CCCGAAAGTCGATATACCTAGC
YUC5	1.9	MT-459	TGGAGCTAGTAGACGGTCAG
		MT-460	GAAACGGCGATTTCGGGAAC
YUC8	2.0	MT-271	GGCGGCTTGTCTCCATGAAC

		PH-171	GATGAACTGACGCTTCGTCG
YUC9	2.0	MT-297	GCTAACCACAATGCAATTAC
		MT-298	CATCACTGAGATTCCAAATG
IAA29	1.94	MT-157	CTTCCAAGGGAAAGAGGGTGA
		MT-158	TTCCGCAAAGATCTTCCATGTAAC
BR6ox2	2.05	oVCG-740	GTGAGCGGTTCGTCAGGTC
		oVCG-741	GGTAACGATCTTGTATTCCGG
CPD	2.06	oVCG-726	GCACTTTCAACCCTTGGAGA
		oVCG-727	CAGAGAGTGCAACCCTAGCC
HSP17.6B	2.21	YI578	CAGGTTAAGGCTGCGATGGA
		YI579	AGCCTTAGGCACCGTAACAG
GA3OX1	1.88	YI622	TACCGACTCCACCCTCCTAA
		YI623	GACCCAACCAAGATCATCGC
SAUR22	1.99	MT515	GTATGAGAGTGGCACTAAG
		MT516	GCTCTGGTGAGAAGTCTAC

**Oligos used for ChIP-qPCR**			
**Target**	**Primer Efficiency**	**Oligonucleotide**	**Sequence**
IAA29 peak (G-box)	1.86	MK54	ACATTACGCCACGAGTAG
		MK55	GATCAACCAAGCAGAAGAG
IAA29 control	1.92	MK60	GGGATGTTACATGGAAGTAAG
		MK61	ATGAACAGATTCCGCAAAG
YUC8 peak (G-box)	1.97	oASF213	GGAATGGGTTTGATGTGGAA
		oASF214	GATTCTTTGTGGGACCAACG
YUC8 control	2.03	MK34	AGCTGGCCTATGAAATAAC
		MK35	AGTGGACGATCAATTCTC

**Oligos used for cloning (Two-hybrid vectors)**			
**Plasmid**	**Strategy**	**Oligonucleotide**	**Sequence**
pAD-PIF7 (pVG20)	Digestion of amplified fragment and pGADT7 with EcoRI and BamHI followed by T4 ligation.	oVCG-193 (EcoRI)	TGAATTCCAaTCGAATTATGGAGTTAAAGAG
		oVCG-194 (BamHI)	CGATGGATCCCCTAATCTCTTTTCTCATGA
pAD-PIF4 (pVG22)	Blunt T4 ligation of amplified fragment into SmaI digested pGADT7	33265	GAACACCAAGGTTGGAGT
		33183	CTAGTGGTCCAAACGAGAAC
pBD-PIF7 (pASF13)	InFusion cloning between NcoI-linearized pGBKT7 and PCR amplified PIF7/PIF4.	oASF205	AGGACCTGCATATGGCCATGTCGAATTATGGAGTTAAAGAGCTCACA
		oASF206	CCGGGAATTCGGCCTCCATGCTAATCTCTTTTCTCATGATTCGAAGAACTTGAAG
pBD-PIF4 (pASF14)		oASF207	AGGACCTGCATATGGCCATGGAACACCAAGGTTGGAGTTTTG
		oASF208	CCGGGAATTCGGCCTCCATGCTAGTGGTCCAAACGAGAACCG
